# Complete Genome Sequence of Cluster C Mycobacteriophage McGee

**DOI:** 10.1128/mra.00768-22

**Published:** 2022-09-08

**Authors:** Imade Y. Nsa, Ayodeji A. Odunsi, Chioma V. Okorie, Seun D. Bamisaye, Rashidat O. Abass, Dennis O. Amadi, Joshua E. Ebitigha, Oluwafemi O. Kadiri, Temidayo M. Makinwa, Solape O. Olaiya, Josephine O. Oluwafemi, Tomilayo L. Owoeye, Zainab A. Sekoni, Abisoye F. Olubajo, Tenny O. G. Egwuatu, Ganiyu O. Oyetibo, Matthew O. Ilori

**Affiliations:** a Department of Microbiology, Faculty of Science, University of Lagos, Lagos, Nigeria; b Institute of Maritime Studies, University of Lagos, Lagos, Nigeria; Queens College CUNY

## Abstract

Mycobacteriophage McGee is a myovirus isolated from a wet soil sample collected at Manassas, VA, using Mycobacterium smegmatis mc^2^155. McGee has a genome 156,008 bp long, containing 237 protein-coding genes, 31 tRNA genes, and 1 transfer-messenger RNA (tmRNA) gene. McGee shares high gene content similarity to phages in actinobacteriophage cluster C1.

## ANNOUNCEMENT

**FIG 1:**
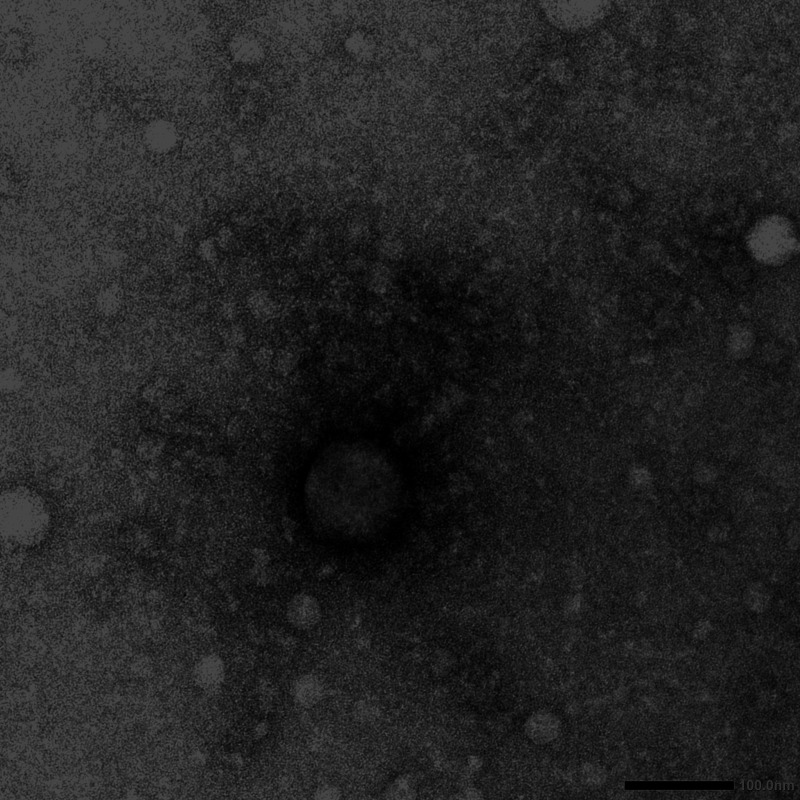
Transmission electron micrograph of McGee. Phage lysate was mounted on a Formvar copper grid and stained with 1% uranyl acetate. McGee has a *Myoviridae* morphotype, with a capsid 103 nm in diameter and a tail 86 nm long (*n* = 1).

Numerous bacteriophages capable of infecting Mycobacterium smegmatis mc^2^ 155 have been isolated and characterized, providing important insights into viral diversity and population structure (1). Here, we report on McGee, a mycobacteriophage newly isolated from soil collected in Manassas, VA (coordinates 38.660592 N, 77.436041 W), using standard procedures (https://seaphagesphagediscoveryguide.helpdocsonline.com/home; accessed 15 July 2022). The soil sample was washed with 7H9 liquid medium, the wash filtered (pore size, 0.02 μm), and the filtrate plated in soft agar overlay containing Mycobacterium smegmatis mc^2^ 155. McGee was purified with multiple rounds of plating. Negative-stain transmission electron microscopy revealed McGee to possess a *Myoviridae* morphology (Fig. 1).

DNA was isolated using the Promega Wizard DNA cleanup kit, prepared for sequencing using the NEB Ultra II library kit (New England Biolabs, Ipswich, MA), and sequenced on an Illumina MiSeq instrument (v3 reagents), resulting in 77,248 single-end 150-bp reads providing 73× coverage. The raw reads were assembled and assessed using the programs Newbler v2.9 (2) and Consed v23 (3), respectively. The assembly yielded a circularly permuted genome of 156,008 bp with 64.6% GC content.

Genes were identified and annotated using DNA Master v5.23.6 (http://cobamide2.bio.pitt.edu/) (4), GLIMMER v3.02 (5), GeneMark v2.5 (6), and the Starterator v463 database (http://phages.wustl.edu/starterator), whereas gene functional assignments were determined using BLASTp searches against the NCBI nonredundant v2.13.0 (7) and actinobacteriophage databases (8), HHpred (9), and Phamerator (10). Aragorn v1.2.41 (11) and tRNAscan v2.0.9 (12) were used to call tRNAs and transfer-messenger RNAs (tmRNAs). Membrane proteins were identified using SOSUI v1.1 (13–15) and TMHMM v2.0 (16, 17). All software was run using default parameters.

A total of 269 putative genes were identified in McGee, including 237 protein-coding genes, 31 tRNAs, and 1 tmRNA. Among those that could be assigned a function, the virion structure and assembly genes include a major capsid protein (gp101), capsid decoration protein (gp103, gp117), tail sheath protein (gp129), tail assembly chaperones (gp132, gp133), tape measure protein (gp134), minor tail proteins (gp138, gp146, gp147), and baseplate wedge protein (gp141, gp142). The genes involved in McGee’s DNA metabolism include DNA helicase (gp188), thymidylate kinase (gp197), DnaC-like helicase loader (gp201), DnaB-like double-stranded DNA (dsDNA) helicase (gp202), DNA primase (gp204), DnaJ-like chaperonin (gp206), single-stranded DNA (ssDNA) binding protein (gp207), DnaE-like DNA polymerase III α (gp208), RF-1 peptide chain release factor (gp209), RecA-like DNA recombinase (gp210), Holliday junction resolvase (gp210), RusA-like resolvase (gp214), Ro-like RNA binding protein (gp233), and multiple proteins with a helix-turn-helix DNA binding domain (gp54, gp61, gp62, gp196). Genes involved in lysis, lysin A, holin, and lysin B are encoded by gp252, gp253, and gp254, respectively. The largest gene in McGee is a hypothetical protein gp98 (4,113 bp long) and the smallest gp64 (75 bp long).

Based on its gene content similarity (GCS) of at least 35% to phages in the Actinobacteriophage database (https://phagesdb.org/), McGee was assigned to phage cluster C1 (8, 18, 19). McGee shares the highest GCS with Kboogie (95.98%) and encodes 3 additional HNH endonucleases (gp148, gp221, gp251) relative to Kboogie.

### Data availability.

The complete genome sequence for McGee can be found at GenBank under accession number ON637764, while its raw reads have been deposited under Sequence Read Archive accession number SRX14483217.
